# Supplementary material for: The neutral rate of whole-genome duplication varies among yeast species and their hybrids
Source: Nat Commun. 2021 May 25;12:3126. doi: 10.1038/s41467-021-23231-8 (PMC8149824; doi:10.1038/s41467-021-23231-8)
Supplement: Supplementary file 1 — Supplementary Information [file 41467_2021_23231_MOESM1_ESM.pdf]

Supplementary Information

The neutral rate of whole-genome duplication varies among yeast species and their hybrids

Marsit *et al*.

Correspondence to: souhir.marsit@gmail.com and christian.landry@bio.ulaval.ca

This PDF file includes:

Supplementary Figures 1 to 15
Supplementary Tables 1 to 2
Supplementary References (1-4)

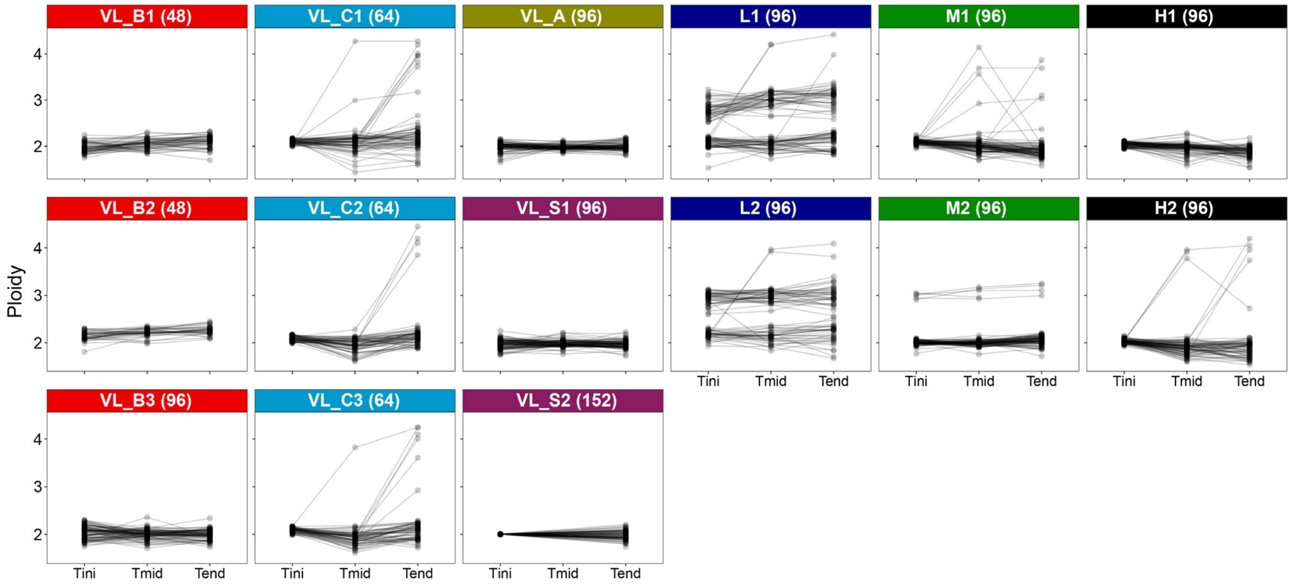

Supplementary Fig. 1 | Ploidy evolution of the 1304 lines from the 15 different crosses. Ploidy was measured at three different generation timepoints, T_{ini} (22 generations), T_{mid} (352 generations) and T_{end} (770 generations and 2062 generations for VL_S2). The numbers in parentheses represent the number of biologically independent lines.

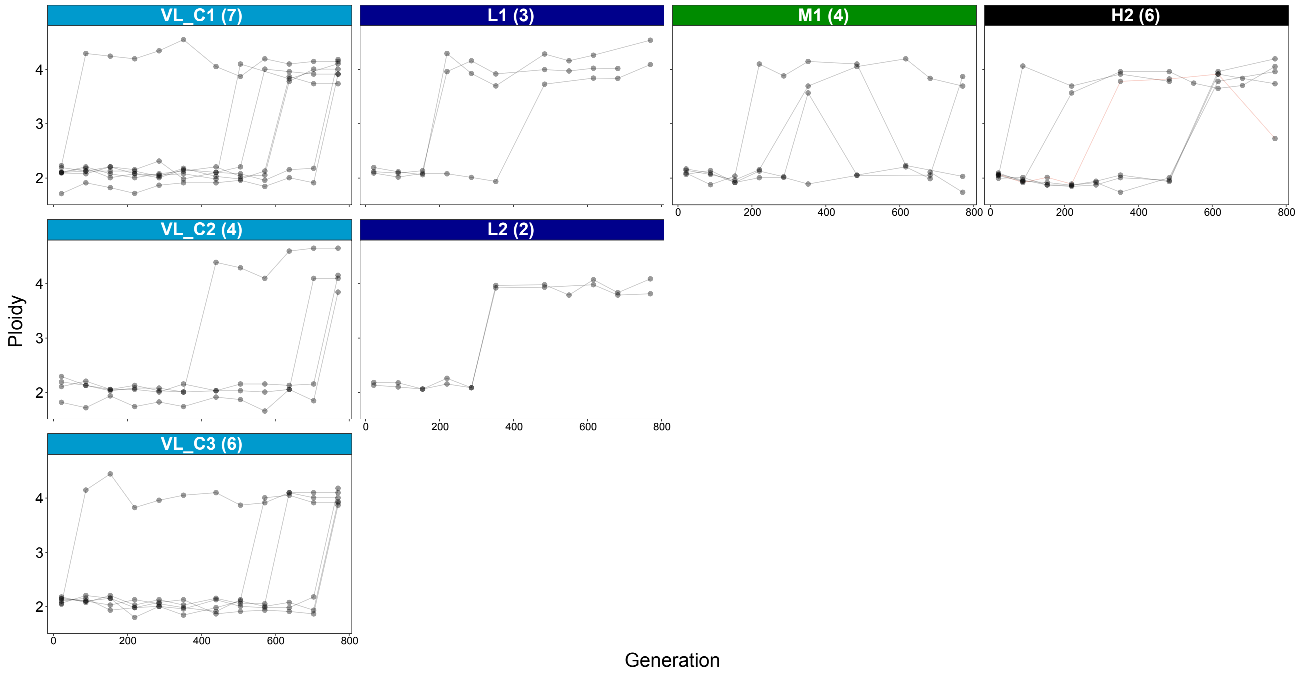

Supplementary Fig. 2 | Whole-genome duplication occurred at different generation timepoints in VL_C intra-lineage crosses and hybrids. Ploidy was measured at ~70 generation intervals for the 32 lines where whole-genome duplication occurred. The H2_43 line is colored in red. The numbers in parentheses represent the numbers of biologically independent lines.

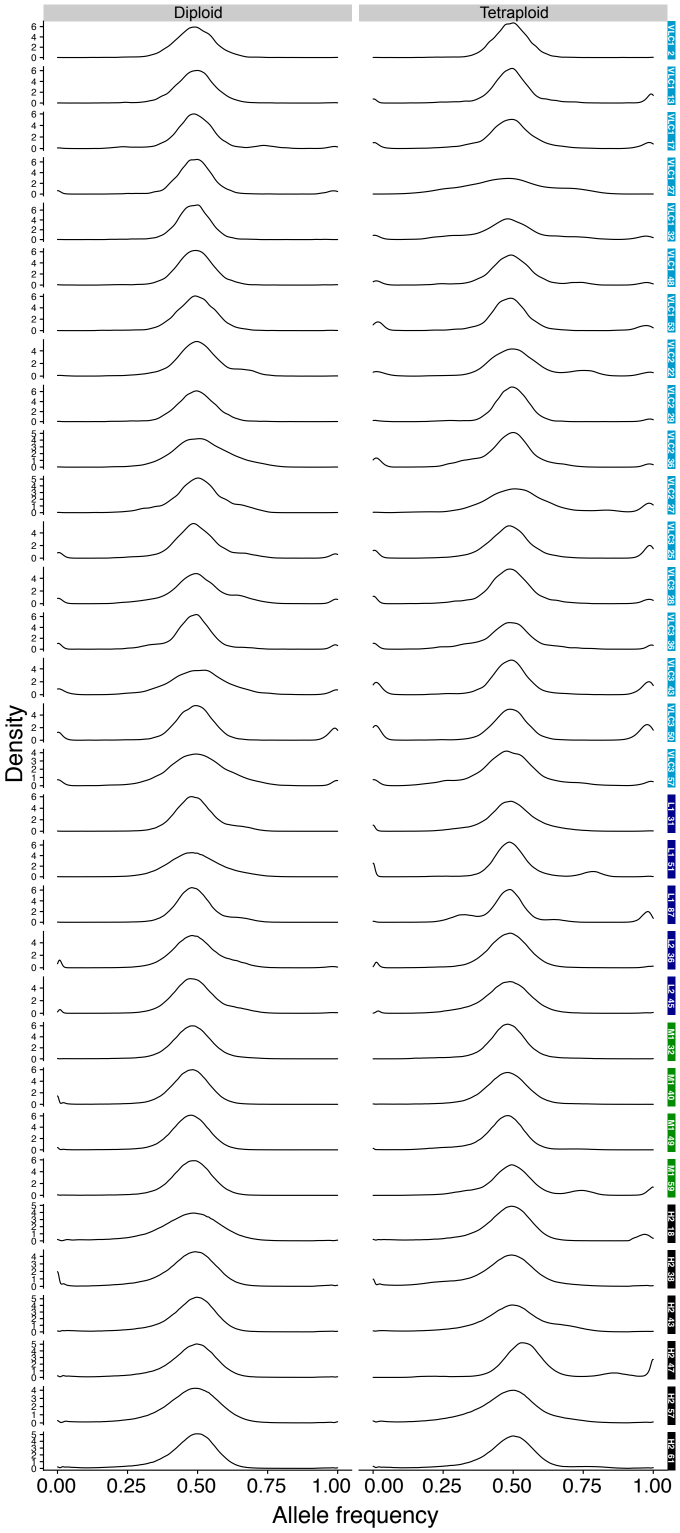

Supplementary Fig. 3 | The identified tetraploid lines result from a whole-genome duplication of both parental genomes. Allele frequencies over the 16 chromosomes of the 32 tetraploid independent lines that went through whole-genome duplication at their diploid and tetraploid state confirm that both parental genomes have been duplicated.

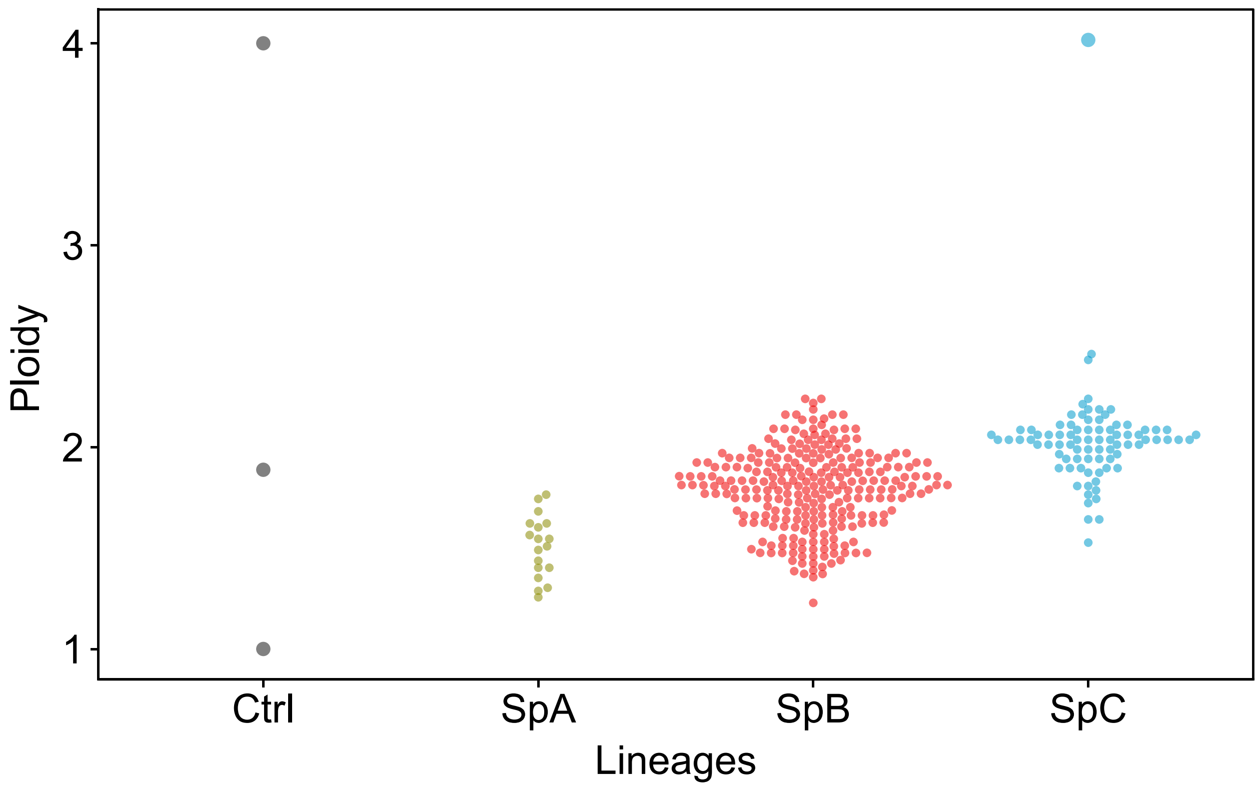

Supplementary Fig. 4 | A tetraploid *SpC* natural isolate is identified among natural *S. paradoxus* strains. The ploidy of 366 wild North-American *S. paradoxus* isolates from *SpA* (n=18), *SpB* (n= 265) and *SpC* (n= 83) lineages was estimated using flow cytometry (n represent the number of independent wild strains). Ctrl corresponds to MSH604 *SpB* parent at haploid and diploid states used as controls.

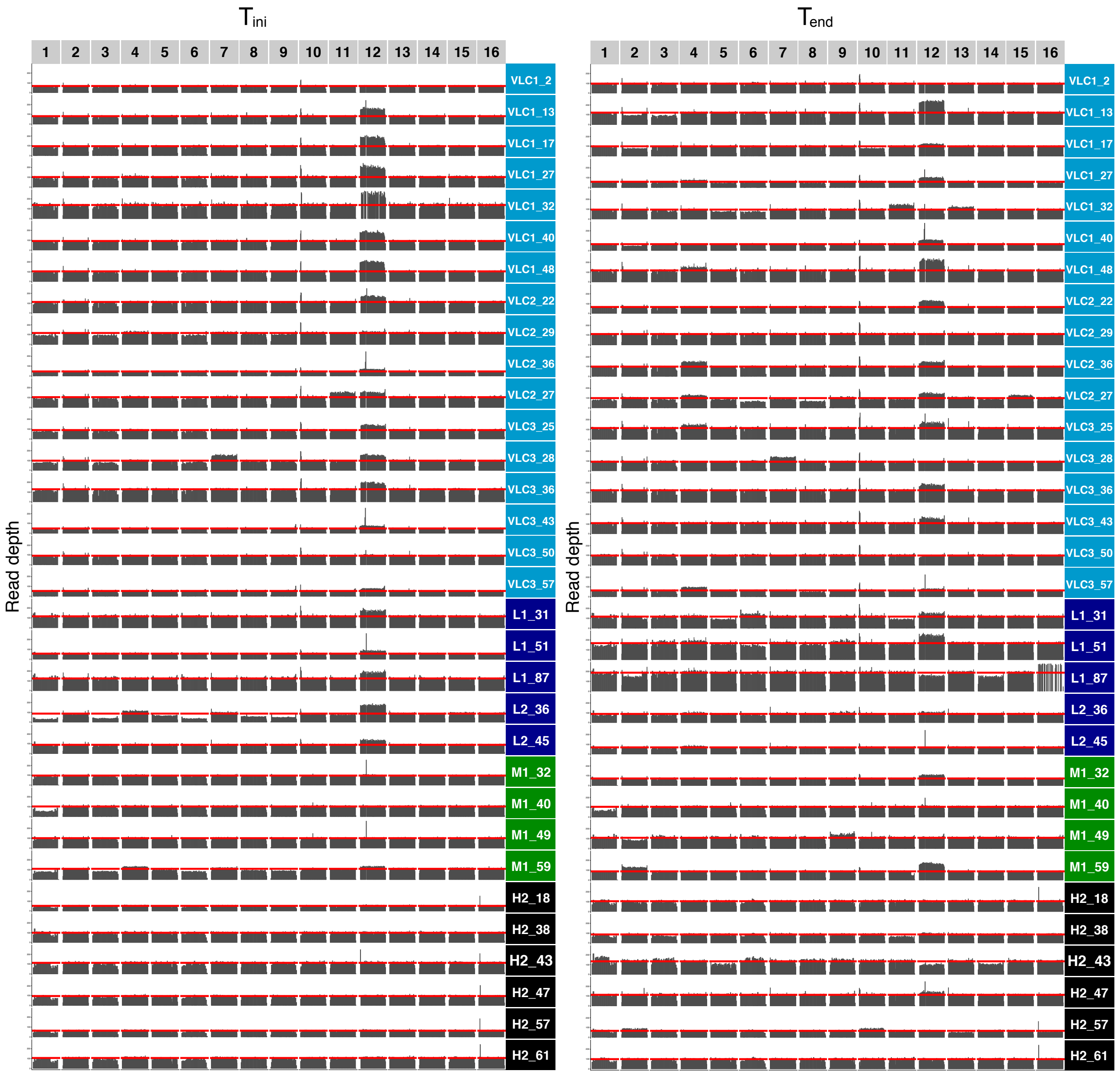

Supplementary Fig. 5 | Several aneuploidies are detected in tetraploid lines. Sequencing read depth over the 16 chromosomes of the 32 independent lines where whole-genome duplication occurred following mating (T_{ini}, diploids) and at the end of the experiment (T_{end}, tetraploids) reveal several aneuploidies. Black bars correspond to the read depth over windows of 10 kb across chromosomes and the red lines correspond to the average coverage of the whole genome.

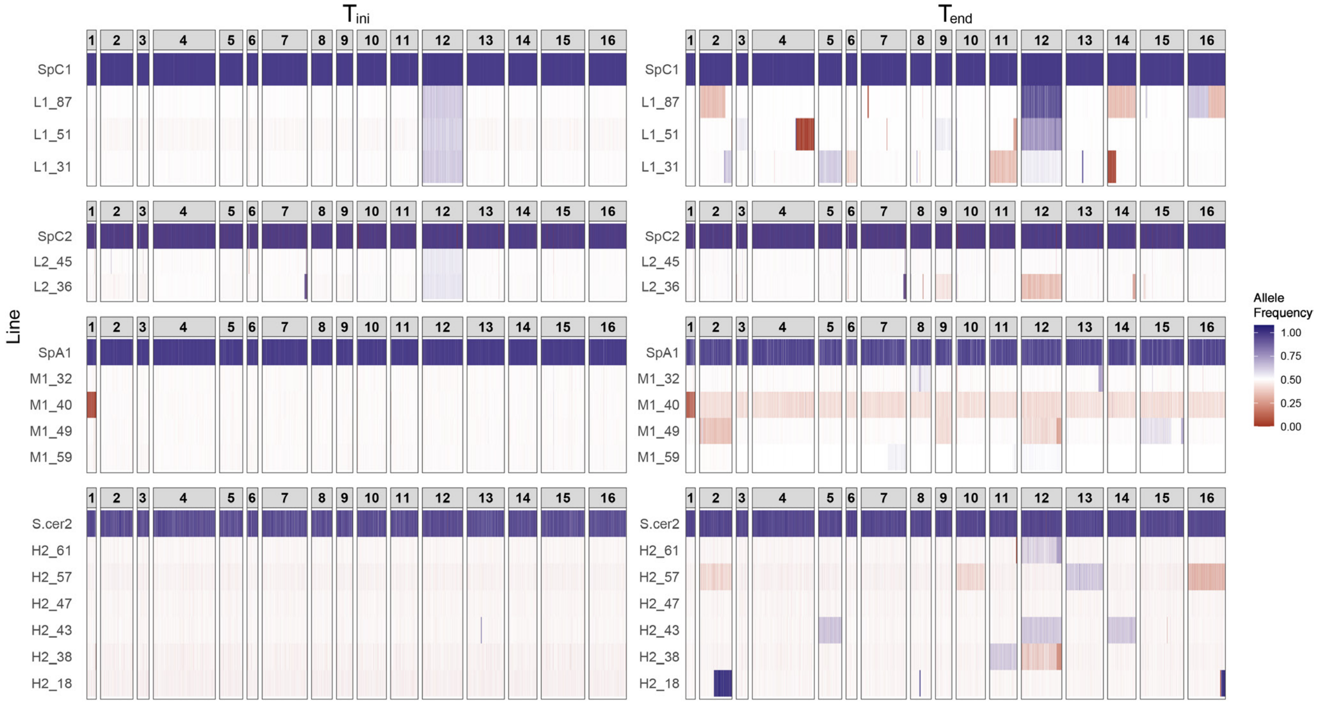

Supplementary Fig. 6 | Several loss of heterozygosity events are detected in tetraploid hybrids. Within genome allele frequencies over the 16 chromosomes in tetraploid hybrids reveal many LOH events and variation in parental contributing allele frequencies resulting from aneuploidies following mating (T_{ini}) and at the end of the experiment after 770 generations (T_{end}).

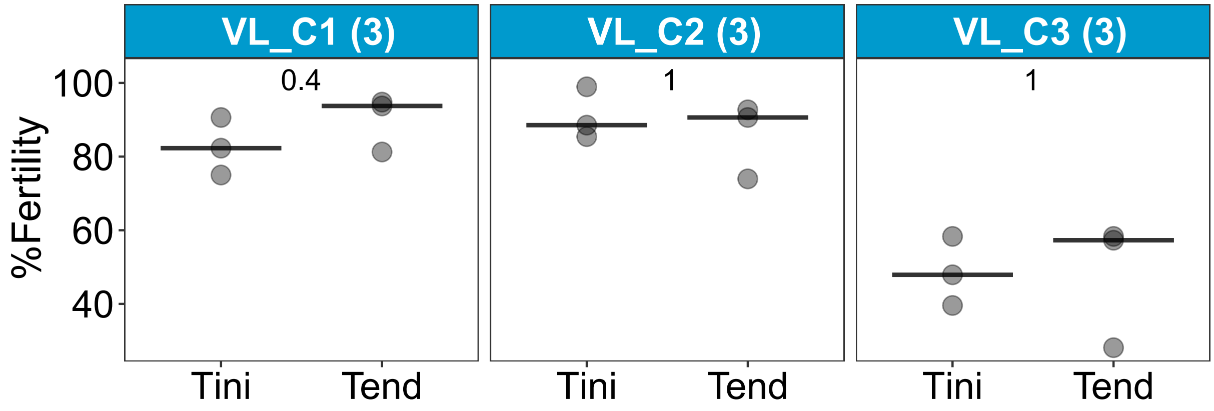

Supplementary Fig. 7 | There is no systematic trend towards fertility gain or loss during neutral evolution in intra-lineage VL_C crosses diploid lines. The fertility of F1 hybrids from diploid intra-lineage VL_C crosses was examined following mating (T_{ini}) and at the end of the experiment (T_{end}). Fertility here is measured using as proxy the percentage of viable spores after meiosis. Medians are shown by horizontal bars. *P* values from a paired two-sided t-test are shown. The numbers in parentheses represent the number of biologically independent lines.

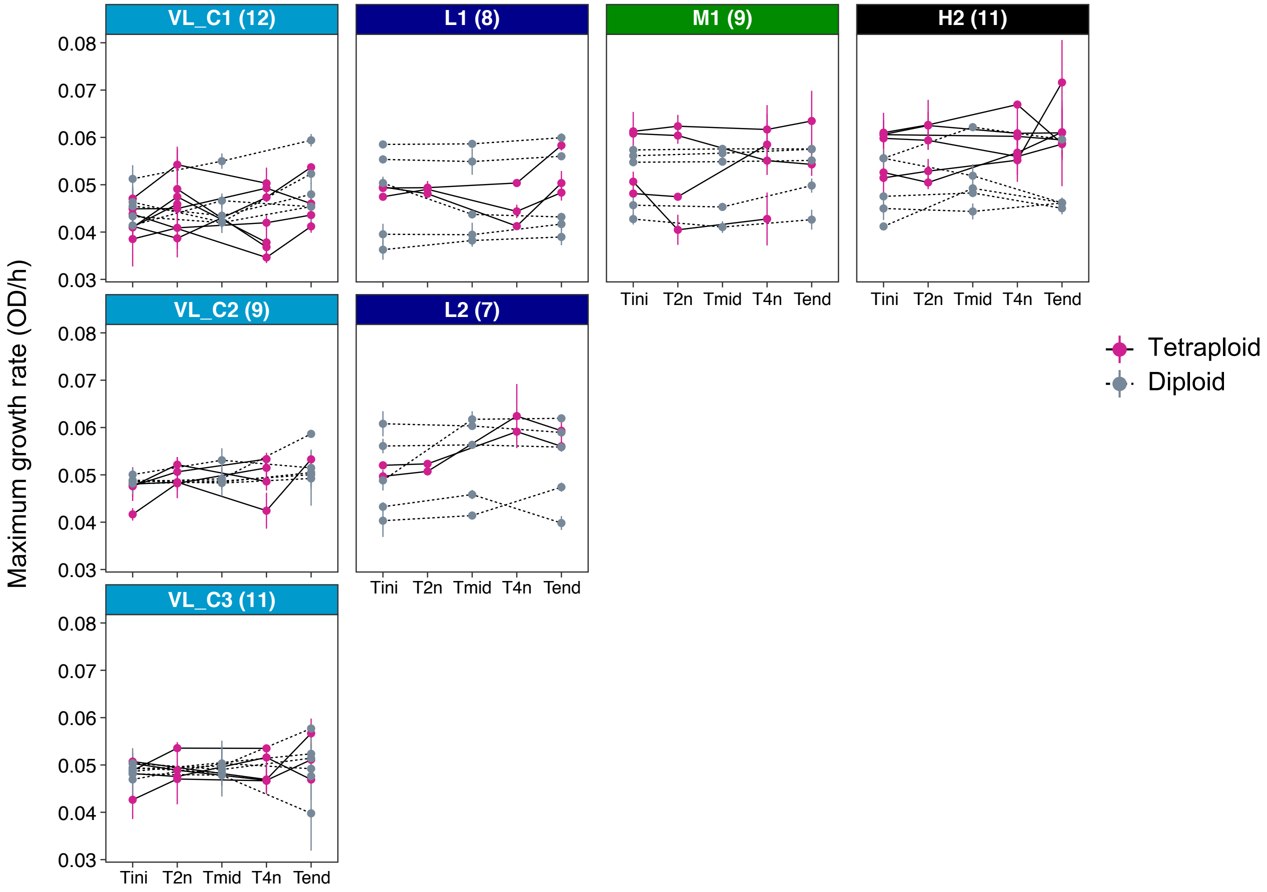

Supplementary Fig. 8 | Whole-genome duplication does not lead to systematic fitness gain or loss. The maximum growth rate of 32 tetraploids at 4 timepoints and 35 diploids randomly selected at 3 time points was measured. The maximum growth rate of tetraploids was measured following mating (T_{ini}), before whole-genome duplication (WGD) (T_{2n}), after WGD (T_{4n}), and at the end of the experiment after 770 generations (T_{end}). Maximum growth rate of diploids was measured following mating (T_{ini}), at the middle of the experiment after 352 generations (T_{mid}) and at the end of the experiment after 770 generations (T_{end}). Four to five independent replicates were performed for each line. The center point represents the mean and error bars represent the mean plus and minus the standard deviation. The numbers in parentheses represent the numbers of biologically independent lines.

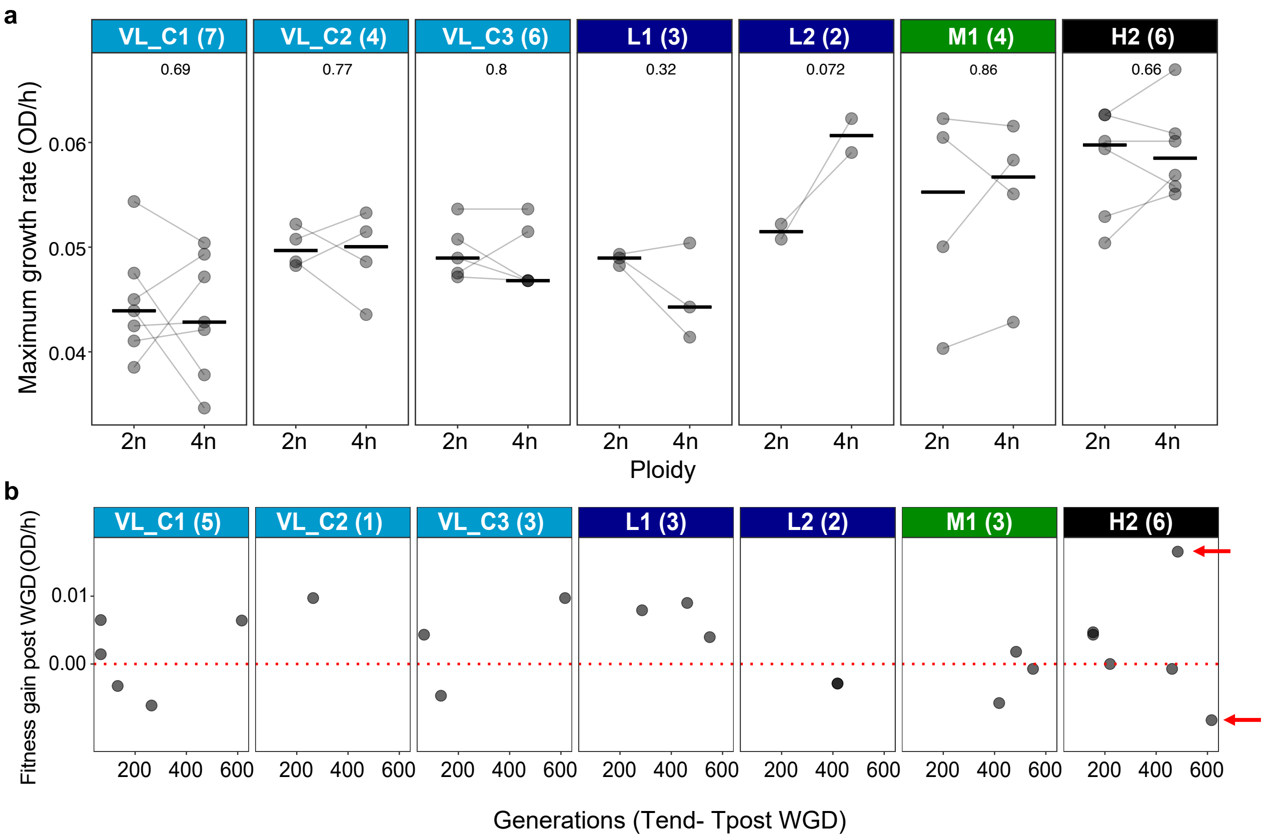

Supplementary Fig. 9 | Tetraploid lines show gains and losses of fitness following Whole-genome duplication and at the end of the experiment. **a** Maximum growth rate before (2n) and after whole-genome duplication (WGD) (4n) of intra-lineage VL_C crosses and hybrids. *P* values from a two-sided paired t-test are shown above. Medians are shown by horizontal bars. **b** Fitness gain of tetraploids at the end of the experiment. Fitness gain post WGD is calculated as the difference of the maximum growth rate between the end of the experiment and following WGD. Evolved generations are calculated as the difference between the number of generations following WGD (Tpost WGD) and the end of the experiment (T_{end}). Red arrows indicate the H2_43 and H2_57 lines, showing respectively, markedly increased and decreased fitness. The numbers in parentheses represent the number of biologically independent lines.

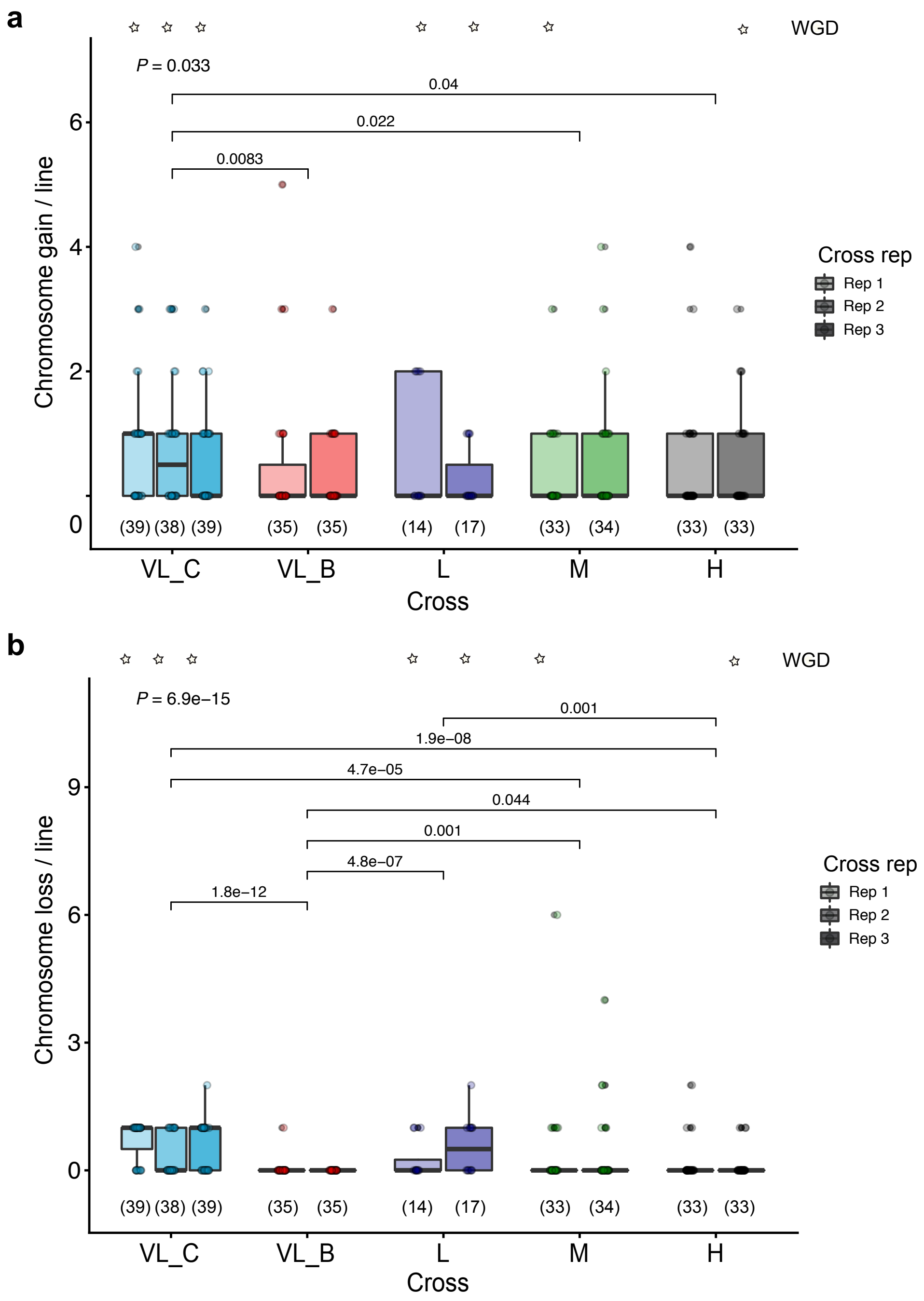

Supplementary Fig. 10 | Chromosome gain and loss in intra-lineage crosses and hybrids. **a** Chromosome gain rate in intra-lineage crosses and hybrids. **b** Chromosome loss rate in intra-lineage crosses and hybrids. *P* values from Kruskal Wallis test (above) and pairwise two-sided Mann–Whitney–Wilcoxon tests are shown (only *P* values <0.05 are shown). The crosses with a star are those where whole-genome duplication occurred. The numbers in parentheses represent the number of biologically independent lines. For all boxplots the bold center line corresponds to the median value, the box boundaries correspond to the 25th and the 75th percentile, the whiskers correspond to 1.5 times the interquartile range, minimum and maximum values correspond to the minima and maxima and the dots correspond to the individual data points.

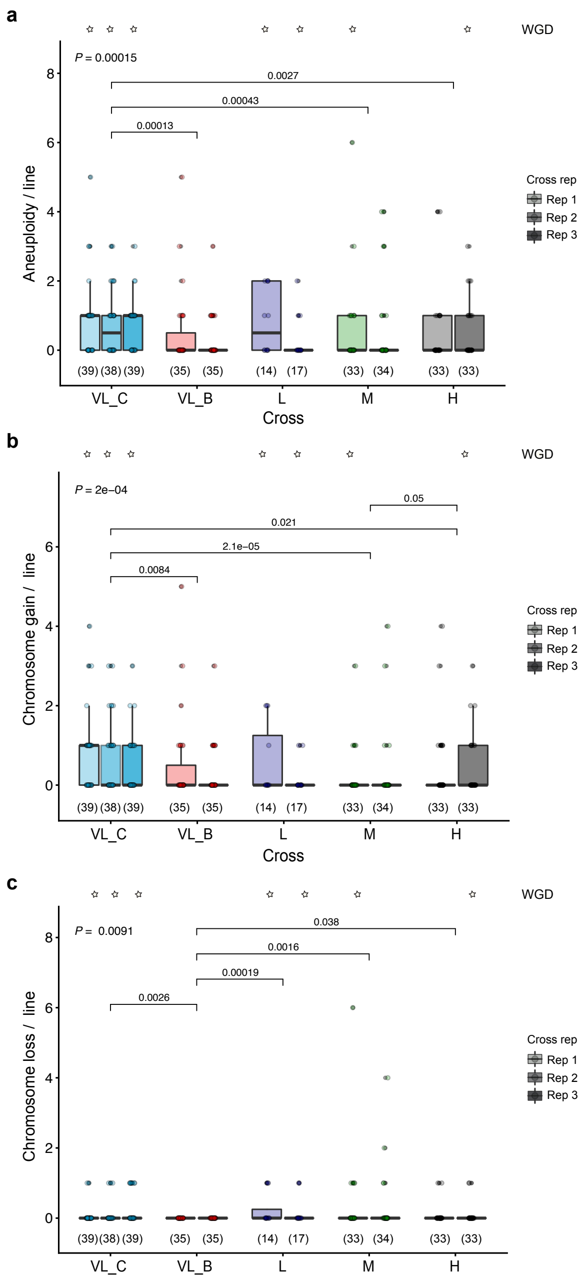

Supplementary Fig. 11 | Chromosome gain and loss in intra-lineage crosses and hybrids including all chromosomes except chromosome 12. a Aneuploidy rate excluding chromosome 12 in intra-lineage crosses and hybrids. **b** Chromosome gain rate excluding chromosome 12 in intra-lineage crosses and hybrids. **c** Chromosome loss rate excluding chromosome 12 in intra-lineage crosses and hybrids. Numbers in parentheses represent sample sizes. *P* values from Kruskal Wallis test (above) and pairwise two-sided Mann–Whitney–Wilcoxon tests are shown (only *P* values <0.05 are shown). The crosses with a star are those where whole-genome duplication occurred. The numbers in parentheses represent the numbers of biologically independent lines. For all boxplots the bold center line corresponds to the median value, the box boundaries correspond to the 25th and the 75th percentile, the whiskers correspond to 1.5 times the interquartile range, minimum and maximum values correspond to the minima and maxima and the dots correspond to the individual data points.

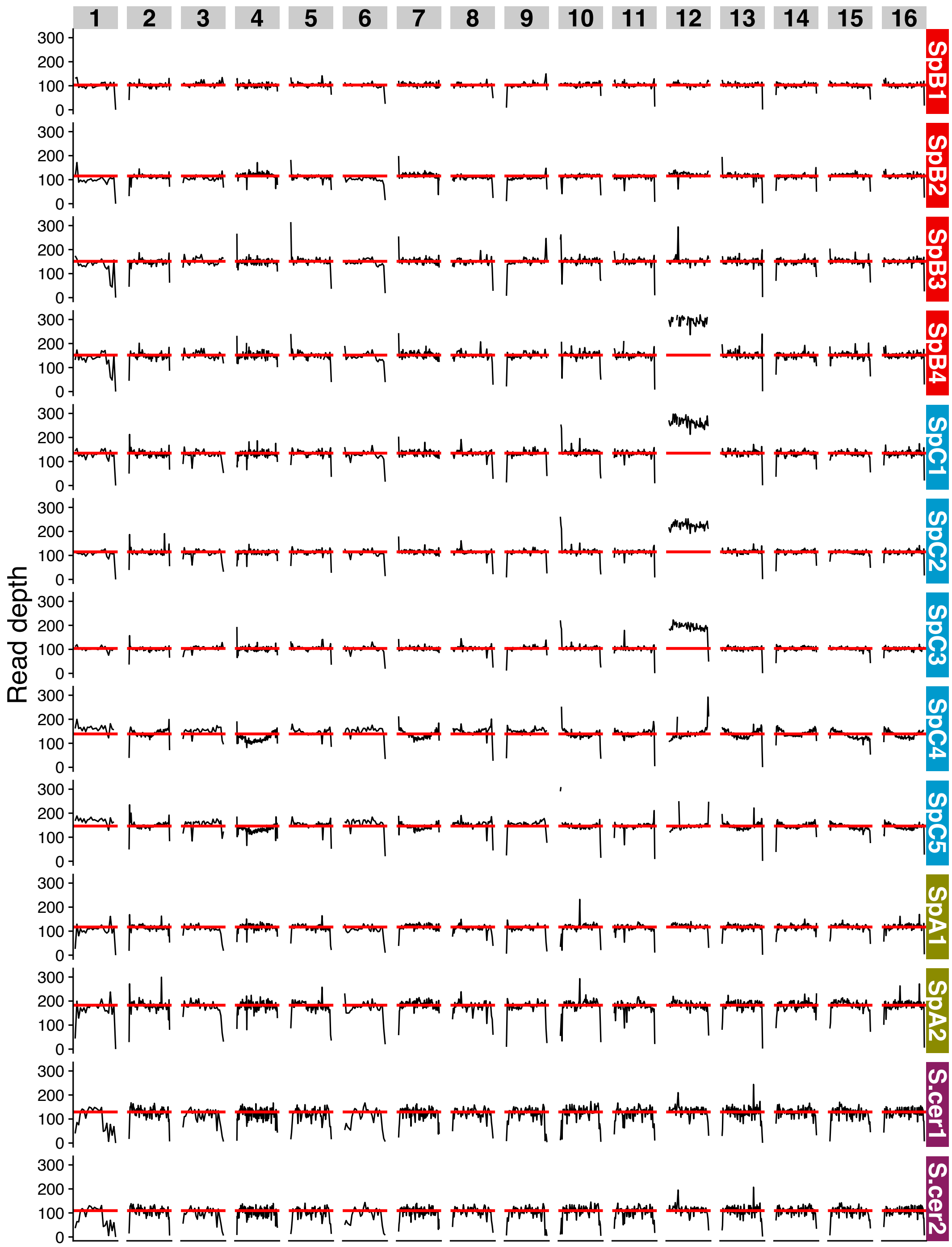

Supplementary Fig. 12 | Several *SpC* parents have an additional copy of chromosome 12. Sequencing read depth over the 16 chromosomes of parental strains used for intra-lineage and inter-specific crosses show an additional copy of chromosome 12 in three *SpC* and one *SpB* parents. Black lines correspond to the read depth over windows of 10kb across chromosomes and the red lines correspond to the average coverage of the whole genome.

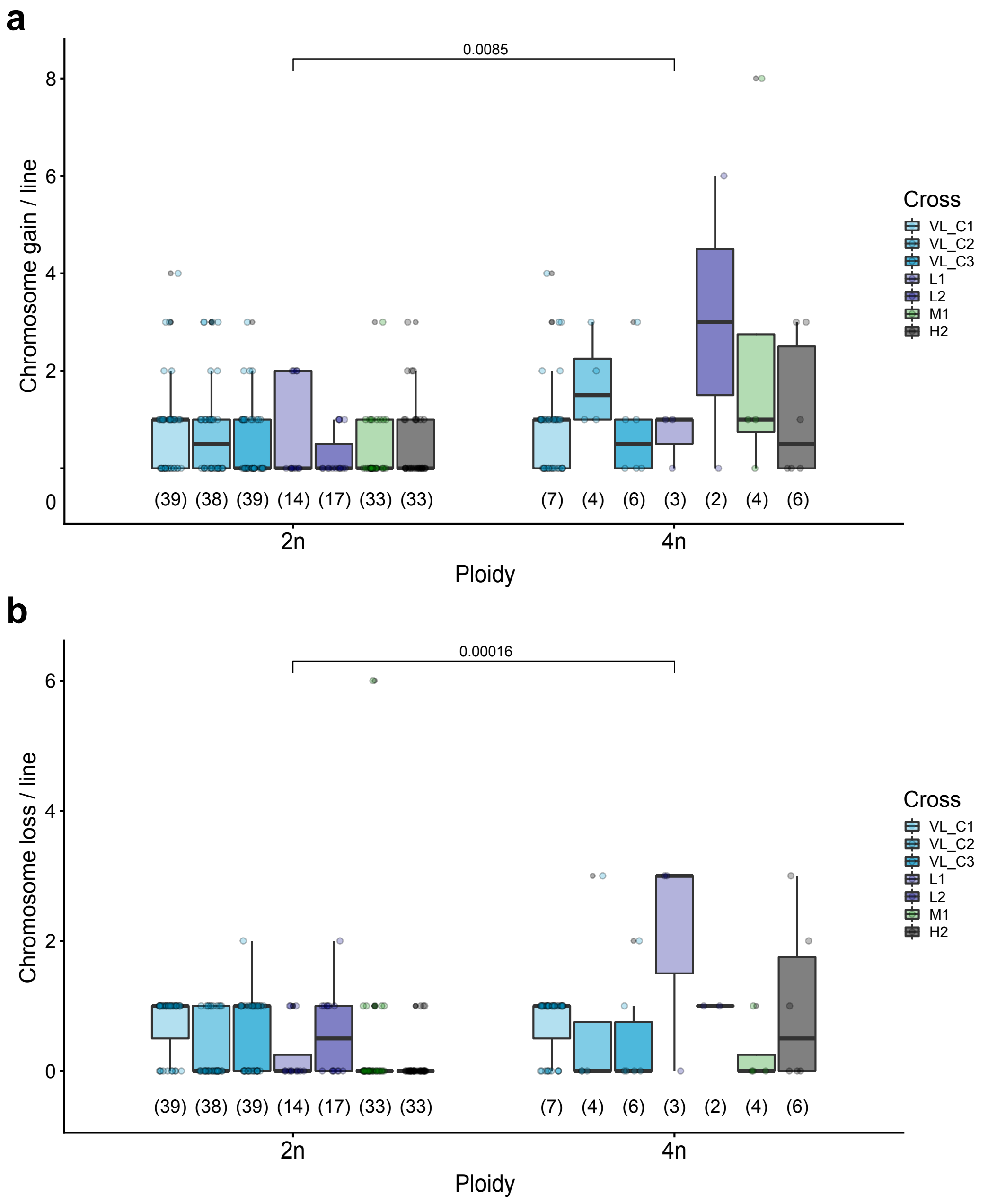

Supplementary Fig. 13 | Chromosome gain and loss rate comparison between diploid and tetraploid intra-lineage VL_C crosses and hybrids. **a** Chromosome gain rate comparison between diploid and tetraploid intra-lineage VL_C crosses and hybrids. **b** Chromosome loss rate comparison between diploid and tetraploid intra-lineage VL_C crosses and hybrids. *P* values from two-sided Mann–Whitney–Wilcoxon test are shown above. The numbers in parentheses represent the number of biologically independent lines. For all boxplots the bold center line corresponds to the median value, the box boundaries correspond to the 25th and the 75th percentile, the whiskers correspond to 1.5 times the interquartile range, minimum and maximum values correspond to the minima and maxima and the dots correspond to the individual data points.

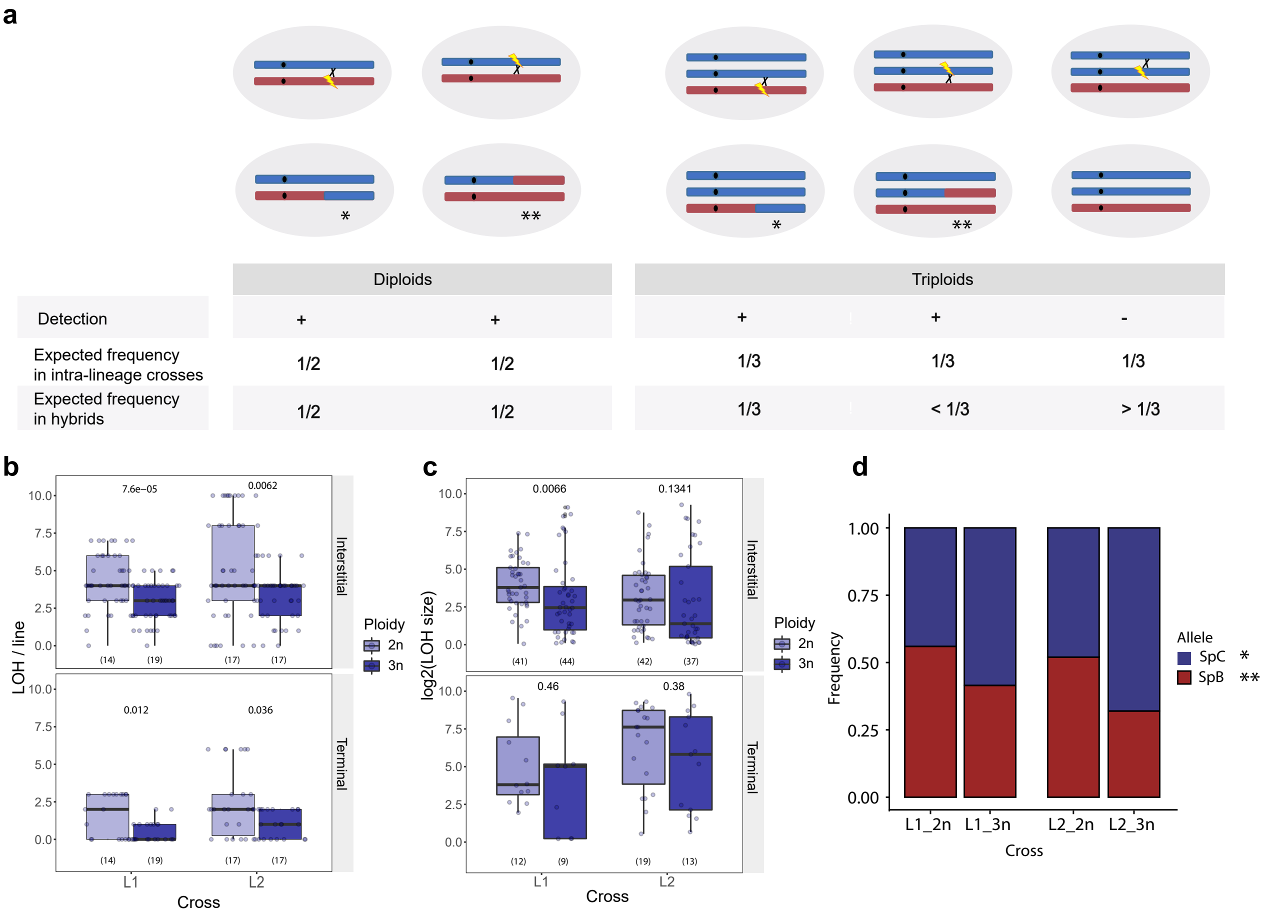

Supplementary Fig. 14 | The presence of two identical copies of chromosomes in allopolyploids likely leads to a decrease in mitotic recombination between homeologous chromosomes. a The different LOH types and their expected frequency in triploids compared to diploids. **b** The frequency of detectable LOH in triploid L1 and L2 hybrids is lower than in diploids. **c** LOH segments size in triploids compared to diploids L1 and L2 hybrids. **d** LOH leading to segments with homozygous *SpC* alleles are more frequent than those leading to an increase in *SpB* allele frequency. LOH events leading to segments with *SpC* alleles are labeled with one star (*) and LOH events leading to segments with *SpB* alleles are labeled with two stars (**). Numbers in parentheses represent the number of biologically independent lines (b) or independent LOH segments (c). *P* values from two-sided Mann–Whitney–Wilcoxon test are shown above. For all boxplots the bold center line corresponds to the median value, the box boundaries correspond to the 25th and the 75th percentile, the whiskers correspond to 1.5 times the interquartile range, minimum and maximum values correspond to the minima and maxima and the dots correspond to the individual data points.

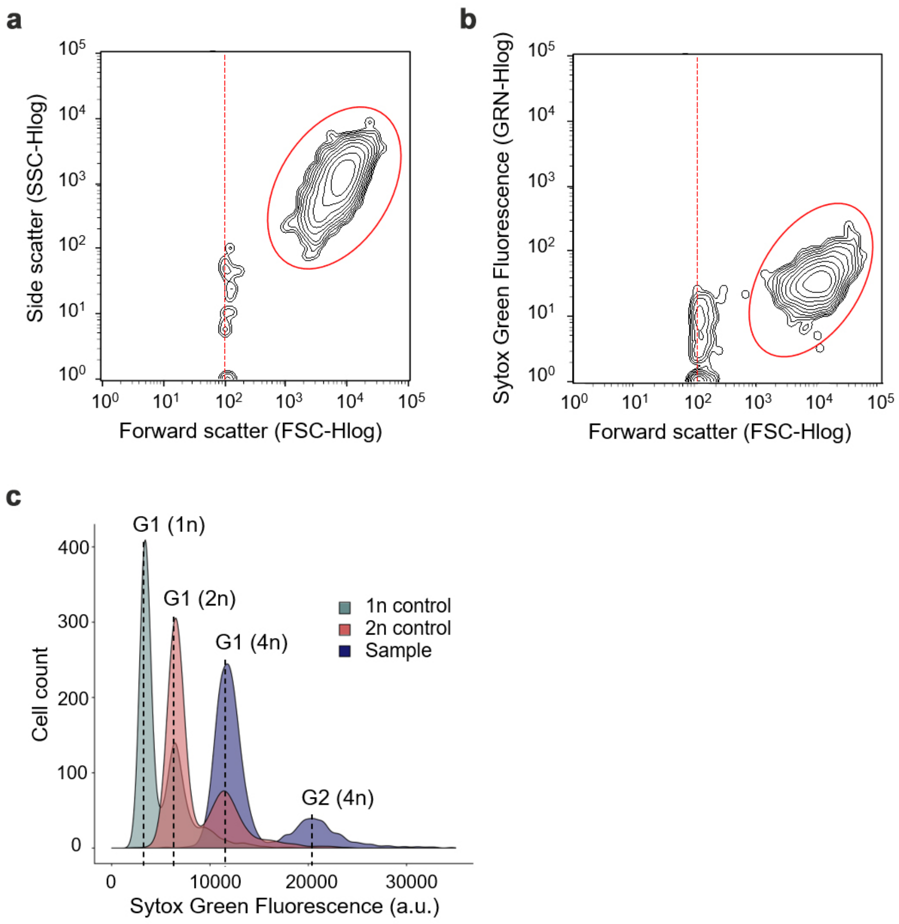

Supplementary Fig. 15 | Flow cytometry gating strategy schematic. Cells were excited with the blue laser at 488 nm. **a** Forward versus side scatter log area plot followed by a **b** forward scatter versus sytox green fluorescence log area plot to remove debris and doublets respectively are shown. **c** The distributions of the sytox green fluorescence values were processed to find the two main density peaks, which correspond to the two cell populations, respectively in G1 and G2 phases using R. Fluorescence values corresponding to the maximum of cell count corresponding to the G1 phase pic were identified. Ploidy estimation was performed by comparing the sample fluorescence values with those of the parental strain *S. paradoxus SpB* strain (MSH604) used as control in both its haploid and diploid state.

Supplementary Table 1. List of crosses used in this study

Cross	a strain	a strain resistance	α strain	α strain resistance	Total number	Reference
VL_B1	MSH604	NAT	LL12_028	G418	48	Charron et al., 2019[1]
VL_B2	UWOPS_91_202	G418	LL12_021	NAT	48	Charron et al., 2019[1]
VL_B3	MSH604	NAT	UWOPS_91_202	G418	96	This study
VL_C1	LL11_004	NAT	MSH587	G418	64	Hénault et al., 2020[2]
VL_C2	LL11_009	G418	LL11_001	NAT	64	Hénault et al., 2020[2]
VL_C3	LL11_009	NAT	LL11_012	G418	64	Hénault et al., 2020[2]
VL_A	YPS744	NAT	YPS644	G418	96	This study
VL_S1	LL13_040	G418	LL13_054	NAT	96	This study
VL_S2	BY4741	-	BY4742	-	152	Hall et al., 2008[3]
L1	MSH604	NAT	LL11_004	G418	96	Charron et al., 2019[1]
L2	UWOPS_91_202	G418	LL11_009	NAT	96	Charron et al., 2019[1]
M1	MSH604	NAT	YPS644	G418	96	Charron et al., 2019[1]
M2	UWOPS_91_202	G418	YPS744	NAT	96	Charron et al., 2019[1]
H1	MSH604	NAT	LL13_040	G418	96	Charron et al., 2019[1]
H2	UWOPS_91_202	G418	LL13_054	NAT	96	Charron et al., 2019[1]

Supplementary Table 2. Rates of WGD, aneuploidy, and LOH by generations of intra-lineage crosses and hybrids.

Cross	Ploidy	WGD rate /gen	Aneuploidy rate /gen	Chromosome gain rate /gen	Chromosome loss rate /gen	LOH rate /gen
VL_B1	2n	0	6.88 +/- 16.3 E-04	6.49 +/- 14.6 E-04	0.38 +/- 2.26 E-04	7.23 +/- 4.09 E-03
VL_B2	2n	0	4.97+/- 8.63 E-04	4.97 +/- 8.63 E-04	0	6.95 +/- 3.56 E-03
VL_B3	2n	0	-	-	-	-
VL_A	2n	0	-	-	-	-
VL_S1	2n	0	-	-	-	-
VL_S2	2n	0	1.04 +/- 1.84 E-04 *	0.97 +/- 0.18 E-04 *	0.7 +/- 0.04 E-05 *	-
VL_C1	2n	1.42E-04	2.02 +/- 1.4 E-03	10.3 +/- 1.28 E-04	9.94 +/- 5.91 E-04	-
VL_C2	2n	8.11E-05	1.41 +/- 1.4 E-03	10.2 +/- 1.3 E-04	3.87 +/- 6.14 E-04	-
VL_C3	2n	1.21E-04	1.44 +/- 1.2 E-03	7.2 +/- 12.8 E-04	7.2 +/- 7.4 E-04	-
L1	2n	4.05E-05	1.23 +/- 1.4 E-03	8.91 +/- 13.2 E-04	3.34 +/- 6 E-04	5.47 +/- 2.26 E-03
L2	2n	2.70E-05	1.07 +/- 1.04 E-03	3.57 +/- 6.12 E-04	7.64 +/- 8.6 E-04	6.95 +/- 3.87 E-03
M1	2n	5.41E-05	8.91 +/- 1.7 E-03	4.46 +/- 8.63 E-04	4.46 +/- 14.4 E-04	3.61 +/- 2.16 E-03
M2	2n	0	1.14 +/- 1.8 E-03	7.08 +/- 12.4 E-04	4.33 +/- 11.3 E-04	3.33 +/- 2.01 E-03
H1	2n	0	7.7 +/- 16.4 E-04	6.08 +/- 12.1 E-04	1.62 +/- 5.55 E-04	2.8 +/- 1.56 E-03
H2	2n	8.11E-05	7.7 +/- 12.5 E-04	6.48 +/- 10.6 E-04	1.22 +/- 3.9 E-04	3.23 +/- 1.9 E-03
VL_C1	4n	-	2.02 +/- 1.4 E-03	1.03 +/- 1.28 E-03	9.94 +/- 5.91 E-04	-
VL_C2	4n	-	3.34 +/- 2.56 E-03	2.34 +/- 8.63 E-03	10 +/- 20 E-04	-
VL_C3	4n	-	1.78 +/- 2.3 E-03	1.11 +/- 1.56 E-03	6.68 +/- 11.2 E-04	-
L1	3n	-	1.8 +/- 1.2 E-03	0.56 +/- 6.78 E-03	1.27 +/- 1.1 E-03	3.56 +/- 1.78 E-03
L1	4n	-	3.57 +/- 3 E-03	0.89 +/- 7.72 E-03	2.67 +/- 2.3 E-03	4.01 +/- 1.2 E-03

L2	3n	-	2.91 +/- 3.2 E-03	1.67 +/- 2.21 E-03	1.34 +/- 1.5 E-03	4.06 +/- 2.11 E-03
L2	4n	-	5.35 +/- 5.6 E-03	4.01 +/- 5.67 E-03	1.34 +/- 0 E-03	4.55 +/- 2.29 E-03
M1	4n	-	3.68 +/- 4.8 E-03	3.34 +/- 4.9 E-03	3.34 +/- 6.6 E-04	6.09 +/- 2.92 E-03
H2	4n	-	2.90 +/- 3.5 E-03	1.56 +/- 1.97 E-03	1.34 +/- 1.6 E-03	3.04 +/- 1.21 E-03

* Zhu et al., 2014 [4].

Supplementary References:

1. Charron, G., Marsit, S., Hénault, M., Martin, H. & Landry, C. R. Spontaneous whole-genome duplication restores fertility in interspecific hybrids. *Nat. Commun.* **10**, 4126 (2019).

2. Hénault, M., Marsit, S., Charron, G. & Landry, C. R. The effect of hybridization on transposable element accumulation in an undomesticated fungal species. *Elife* **9**, (2020).

3. Hall, D. W., Mahmoudizad, R., Hurd, A. W. & Joseph, S. B. Spontaneous mutations in diploid Saccharomyces cerevisiae: another thousand cell generations. *Genet. Res.* **90**, 229–241 (2008).

4. Zhu, Y. O., Siegal, M. L., Hall, D. W. & Petrov, D. A. Precise estimates of mutation rate and spectrum in yeast. *Proc. Natl. Acad. Sci. U. S. A.* **111**, E2310–8 (2014).
