# Supplementary material for: The neutral rate of whole-genome duplication varies among yeast species and their hybrids
Source: Nat Commun. 2021 May 25;12:3126. doi: 10.1038/s41467-021-23231-8 (PMC8149824; doi:10.1038/s41467-021-23231-8)
Supplement: Supplementary file 3 — Descriptions of Additional Supplementary Files [file 41467_2021_23231_MOESM3_ESM.pdf]

Description of additional supplementary files

Supplementary Data 1.
Description: Raw data of fertility used in this study

Supplementary Data 2.
Description: Raw data of optical density for growth rate analysis used in this study
